# Rare insights: Atypical MRI features of juvenile SLE

**DOI:** 10.1016/j.radcr.2024.05.092

**Published:** 2024-06-28

**Authors:** Govind Singh Mann, Neeti Gupta, Nitin Jain

**Affiliations:** a Department of Neurology, Sant Parmanand Hospital, Delhi, India; b Department of Radiology, Sant Parmanand Hospital, Delhi, India

**Keywords:** Brain, Children, Autoimmune, Magnetic resonance imaging, Neuroradiology, Pediatric

## Abstract

Patients with systemic lupus erythematosus (SLE) frequently show symptoms of central nervous system involvement, termed neuropsychiatric SLE (NPSLE). Central nervous system (CNS) vasculitis is one of the neurological pathologies seen in CNS lupus. Patients with NPSLE typically present with nonspecific symptoms such as headache and cognitive impairment. Due to a lack of specific neuroradiological findings, diagnosis and management of such patients remain a big challenge. We report a 5-year-old girl who presented with fever and headache as the only neurological symptoms. Magnetic resonance imaging (MRI) of the brain showed focal grey and white matter lesions, suggestive of inflammatory or demyelinating ethology. Even though MR imaging findings may not be diagnostic of CNS lupus vasculitis, the study is routinely performed as a part of initial evaluation in patients with juvenile SLE showing neurological signs and symptoms.

## Introduction

Systemic lupus erythematosus (SLE) is an auto-immune disease, which is referred to as “juvenile SLE” when it presents before the age of 16 years [1]. A few studies demonstrate cerebral parenchymal involvement in upto 75% of patients [2]. Common neurological presentations are headache, psychosis, seizures, or cognitive dysfunction. Vasculitis is encountered in about 10% of cases [3]. The mortality rate is relatively low, but morbidity may be significant and permanent neurological deficits can occur. In patients developing neuropsychiatric manifestations of SLE, CNS vasculitis should be considered [3]. We report a very uncommon presentation of juvenile SLE, with a vasculitic infarct detected on neuroimaging.

## Case description

A 5-year-old girl, previously fit and well, presented with history of cough, fever for 1 week. There was no history of trauma, recent vaccination, stroke-like presentation or any other clinical symptoms or signs of SLE.

On admission to hospital, she was febrile with normal vital observations and blood pressure. Cardiovascular and respiratory system examination was unremarkable. Abdominal examination revealed mild splenomegaly. Neurological examination was unremarkable.

Blood results at presentation showed normal biochemistry but elevated CRP of 16 mg/L (normal 0-5 mg/L), elevated ESR of 24 mm/hr (normal 0-20 mm/hr), elevated LDH of 1889 U/L (normal 230-460 U/L). The complete blood count showed high total leukocyte count of 16010 cu mm (normal 4000-10500 cu mm) with neutrophilic predominance (85%) and low lymphocytic count (12%), along with mild iron deficiency anemia. Ultrasound of abdomen showed mild splenomegaly.

**Fig. 1:**
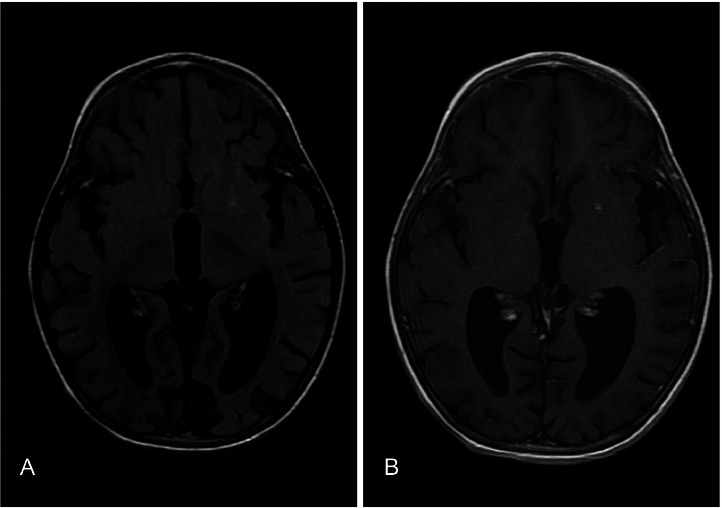
T2 FLAIR (A) and 3D T1 SPGR PC (B) sequence showing a tiny hyperintense focus showing punctate post contrast enhancement in the left putamen, without any perifocal edema.

**Fig. 2:**
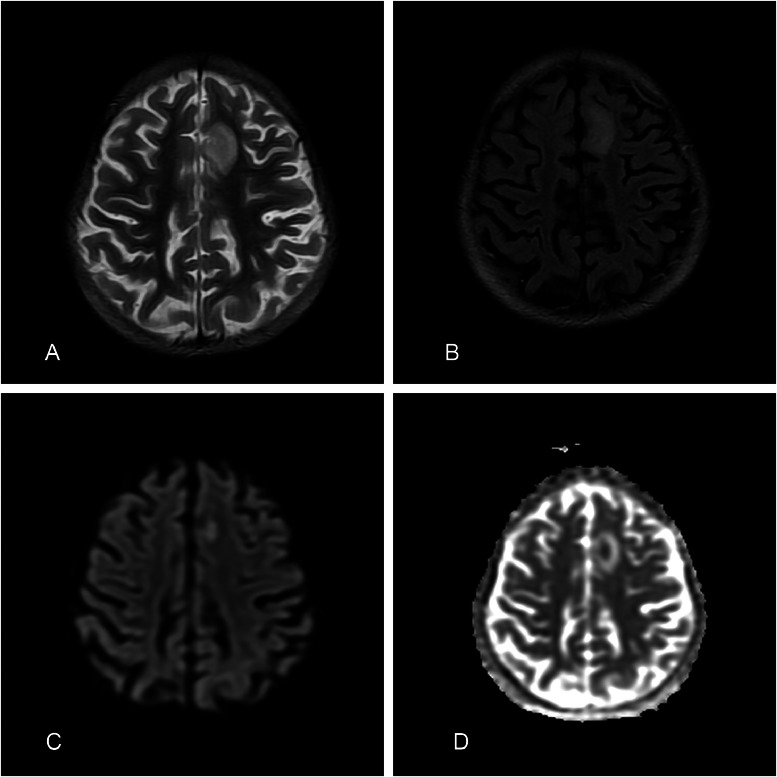
A focal altered signal intensity area is seen involving the cortex and subcortical white matter in left superior frontal gyrus. It appears hyperintense on T2/FLAIR and T2W (A, B), shows mild central diffusion restriction on DWI/ADC images (C, D), with no significant post contrast enhancement.

**Fig. 3:**
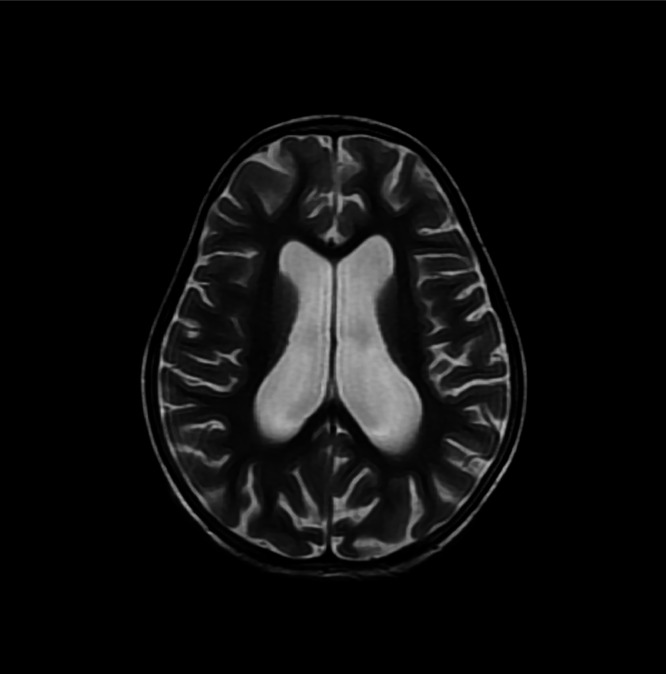
T2W showing generalized volume loss of both cerebral hemispheres.

On day 3 of admission patient developed high grade fever with severe headache and one episode of vomiting. MRI brain was performed which showed a small altered signal intensity, nonenhancing area involving the cortex and subcortical matter in left superior frontal gyrus, showing mild central diffusion restriction on DWI/ADC images, with a small punctate enhancing focus in left putamen on post contrast images (Fig. 1, Fig. 2). Generalized volume loss of both cerebral hemisphere was also noted (Fig. 3). Possibility of demyelinating or inflammatory/ vasculitic etiology was raised on the basis of MRI findings. Later electroencephalogram (EEG) was done and the findings were suggestive of epileptiform discharges with diffuse slowing of left hemisphere. CSF findings were normal. All cultures including blood, CSF and urine were reported as no growth. The virology screen was negative. Echocardiography was normal. ANA titre was positive with nuclear homogenous (AC-1) and cytoplasmic pattern.

At this point rheumatology opinion was sought to rule out auto-immune condition. The C3,C4 levels were low and anti-dsDNA antibody was strongly positive. ANCA levels were normal. She did satisfy the American College of Rheumatology (ACR) criteria for diagnosis of SLE.

The lab markers were suggestive of SLE but no convincing clinical features were found. She was then started on intravenous pulsed Methylprednisolone followed by oral prednisone in tapering doses. The patient had a favorable response to immunosuppressive therapy during her hospital stay. The patient was transferred to a pediatric rheumatologist in a different hospital for further follow up.

## Discussion

Juvenile SLE is a diagnosis of exclusion, achieved by analyzing clinical, laboratory and imaging data [4]. Our patient presented with non-specific symptoms including fever, headache, and vomiting with no clinical findings suggestive of SLE. MR imaging raised a possibility of demyelinating or inflammatory etiology and EEG findings suggesting epileptiform discharges with diffuse slowing of left hemisphere. The diagnosis of SLE was made based on positive immunological markers, anemia, lymphopenia, splenomegaly.

The pathogenesis of juvenile SLE is still incompletely understood but most likely the process is T-cell and autoantibody-mediated damage to neuronal tissue [5]. A study in juvenile SLE reported neuropsychiatric events in 40% of them. Seizures were the most frequent neuropsychiatric manifestation in 50% of patients, followed by headache and depression in 36% of patients, stroke in 26% of patients, chorea in 16% of patients, psychosis and neuropathy in 13% of patients, and myelitis in 6% of patients [6]. SLE vasculitis. Juvenile SLE is associated with small vessel ANCA-negative vasculitis [7].

While MRI is fundamental to rule out alternative diagnosis, there are no definitive neuro-radiological findings for juvenile SLE. Studies report white matter hyperintensities as the most commonly observed abnormality in patients of juvenile SLE [8]. Focal white and grey matter hyperintensities may be attributed to nonspecific histological changes such as gliosis, edema, focal reduced neuronal density, inflammatory infiltration and demyelination [8,9]. MR angiography (MRA) with vessel wall imaging may increase the diagnostic accuracy of vasculitis [7]. The recent European evidence-based recommendations (the SHARE initiative) suggest that MRI along with CSF analysis, EEG, neuropsychological assessment of cognitive function, visual evoked potential (VEP) test and nerve conduction studies should be a part of the initial diagnostic work-up of SLE patients with neuropsychiatric manifestations [10].

MRI is essential for the initial assessment and in therapeutic decision making when the results of other tests are not readily available [2]. Although MRI is sensitive in imaging CNS lesions, the findings are usually nonspecific and not diagnostic of SLE. Therefore the MRI findings should be interpreted along with patient's history, neurological abnormalities, and laboratory tests. MRI may be a more sensitive way of determining clinical outcome compared to other tests, including CSF examination [1].

## Conclusion

The most sensitive non-invasive imaging study for cerebral SLE-related vasculitis is MRI [1,9]. However, MRI findings are usually nonspecific, with white matter hyperintensities as the most commonly observed [8]. Diagnosing neuropsychiatric juvenile SLE remains challenging as clinical data, diagnostic strategies and evidence-based regimens are usually unavailable [10]. We recommend that using imaging techniques that combine morphological with functional imaging may improve the detection rate of CNS involvement in juvenile SLE.

## Patient consent

Written informed consent was obtained from a legally authorized representative for anonymized patient information to be published in this article.
